# Keys to Unravel the Stability/Durability Issues of Platinum-Group-Metal Catalysts toward Oxygen Evolution Reaction for Acidic Water Splitting

**DOI:** 10.1021/acscentsci.4c01363

**Published:** 2024-11-13

**Authors:** Yangdong Zhou, Weijia Guo, Lixin Xing, Zhun Dong, Yunsong Yang, Lei Du, Xiaohong Xie, Siyu Ye

**Affiliations:** † Huangpu Hydrogen Energy Innovation Centre/School of Chemistry and Chemical Engineering, Guangzhou University, Waihuanxi Road 230, Guangzhou 510006, P. R. China; ‡ School of Chemistry and Chemical Engineering, Chongqing University, Daxuecheng South Road 55, Chongqing 401331, P. R. China; § School of Materials Science and Physics/School of Chemical Engineering and Technology, China University of Mining and Technology, Daxue Road 1, Xuzhou 221116, P. R. China; ∥ SinoHykey Technology Company Ltd., Hongyuan Road 8, Huangpu District, Guangzhou 510760, P. R. China

## Abstract

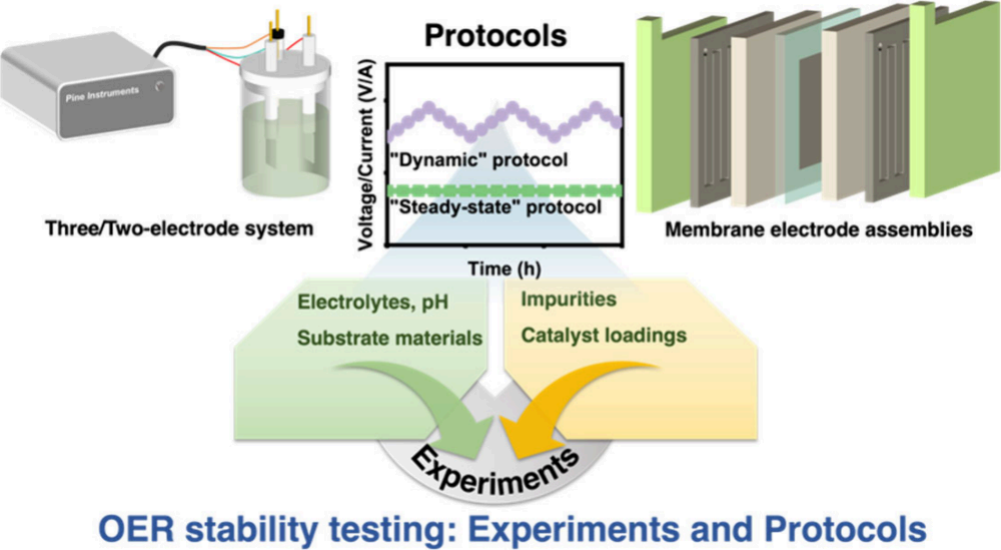

Proton exchange membrane (PEM) water electrolyzers stand as one of the foremost promising avenues for acidic water splitting and green hydrogen production, yet this electrolyzer encounters significant challenges. The primary culprit lies in not only the requirements of substantial platinum-group-metal (PGM)-based electrocatalysts (e.g., IrO_*x*_) at the anode where sluggish oxygen evolution reaction (OER) takes place, but also the harsh high overpotential and acidic environments leading to severe performance degradation. The key points for obtaining accurate stability/durability information on the OER catalysts have not been well agreed upon, in contrast to the oxygen reduction reaction fields. In this regard, we herein reviewed and discussed the pivotal experimental variables involved in stability/durability testing (including but not limited to electrolyte, impurity, catalyst loading, and two/three-electrode vs membrane-electrode-assembly), while the test protocols are revisited and summarized. This outlook is aimed at highlighting the reasonable and effective accelerated degradation test procedures to unravel the acidic OER catalyst instability issues and promote the research and development of a PEM water electrolyzer.

## Abstract 2

The key experimental parameters/protocols as well as their effects on the stability tests of catalysts for acidic oxygen evolution are critically reviewed and discussed in this outlook.

## 1 Introduction

Given its potential to help address the climate crisis and to seek renewable energy supplies, hydrogen energy has raised great interest because of its clean, environmentally friendly, and pollution-free characteristics.^1−3^ Extracting hydrogen from water, i.e., electro-catalyzing water splitting with renewable solar/wind energy, is a clean hydrogen production approach that has been intensively explored recently.^4,5^ Among various techniques for water splitting, the semisolid proton exchange membrane (PEM) water electrolyzer has attracted much attention from industry and academia due to its high current density, high hydrogen purity and fast response to intermissive power inputs.^6−12^

In principle, cathodic hydrogen evolution reactions (HER, 2H^+^ + 2e^–^ → H_2_) and anodic oxygen evolution reactions (OER, 2H_2_O → O_2_ + 4H^+^ + 4e^–^) occur in the PEM electrolyzer stacks, respectively.^1^ Particularly, both reactions require platinum group metals (PGMs) as the catalysts, i.e., Pt for HER and Ir for OER; between them, the anode has a more serious issue, due to more sluggish OER than HER, five-times price of Ir than Pt, and extremely insufficient Ir reserve, leading to a significant obstacle to the widespread deployment and implementation of PEM electrolyzers.^7,13−17^ Thus, decreasing the PGM content particularly at the anode is pursued in this community. Very recently, the Low-PGM (e.g., Ir/RuMO_*x*_)^18−22^ and even PGM-free (e.g., Co/MnO_*x*_)^12,23−26^ catalysts for acidic OER were developed, but the fast degradation of their initial performance under harsh OER is still in concern. This is, at least in part, due to the lack of well-agreed stability/durability test procedures and recognized standards in this field, in contrast to the oxygen reduction reaction (ORR) community.^27−32^ In the past, different research groups used a variety of test parameters, such as electrolytes, pH, catalyst loading and test protocols, etc., setting a barrier to compare published results from different groups—this has made the research and development (R&D) of low-cost acidic OER catalysts challenging and has greatly hindered the development of PEM electrolyzers.

To address these challenges, it is necessary to revisit the diverse protocols (potentio-/galvanostatic and potentiodynamic protocols) and conditions for stability/durability testing as employed by different research groups. For instance, some used chronopotentiometry (*V*–*t*) to evaluate the catalyst stability in an acidic aqueous solution; however, the increased overpotential comes from not only the catalyst degradation but also possible working electrode substrate passivation, material detachment, or oxygen bubble accumulation.^33,34^ Therefore, stating that a catalyst (mostly the reference catalyst in the literature) is not stable using sole chronopotentiometry is not convincible. The study on the stability and durability of the OER catalyst is not routine in material research but should be particularly emphasized and comprehensively conducted with accurate evaluation procedures.

Herein, we briefly review the variables involved in the stability and durability testing process, focusing on the experimental procedures and test protocols documented by various teams. We further delve into the implications of these procedures and protocols on the degradation of the OER electrocatalysts. By adopting reasonable and effective stability/durability test parameters and protocols, the development of long-term stable electrocatalysts will be expedited, thereby promoting the research and development of PEM electrolyzers.

## 2 Key Experimental Parameters for Stability/Durability Study

To evaluate a newly developed OER catalyst, people always test its initial activity and stability/durability in an aqueous solution equipped with two- or three-electrode setups that are convenient, easily operated, and affordable for most research groups. The working electrode is usually prepared by building a homogeneous catalyst film on the rotating disk electrode (RDE) substrates, e.g., glass carbon or gold. This is reasonable, because such a testing configuration benefits the understanding of intrinsic properties. In addition, the H_2_SO_4_ or HClO_4_ solutions are commonly employed to mimic an acidic working condition, encompassing concerns regarding anion adsorption, pH, impurities, etc. Given these considerations, this section will delve into the effects of various parameters. Note that the catalysts have to work in catalyst layers within the membrane electrode assembly (MEA), so that the overall performance degradation of an MEA is contributed by not only the catalyst degradation but also failures of other components. Although the degradation of materials for MEA beyond anode catalysts is out of the scope of this work, we also discuss the gaps between RDE and MEA in this chapter.

### 2.1 Electrolytes

As mentioned above, H_2_SO_4_, HClO_4_, and other acidic electrolytes are usually employed in the aqueous RDE tests to mimic an acidic working environment, which is essential to obtain the intrinsic properties of targeted catalysts. Regarding Pt-based catalyst for ORR, it has been well agreed that HClO_4_ is the best choice due to its negligible adsorption on the catalyst surface.^35,36^ In contrast, for Ir oxide, the benchmark catalysts for acidic OER, it is not well-agreed yet whether the anions bring negative/positive effects or not.

**Figure 1:**
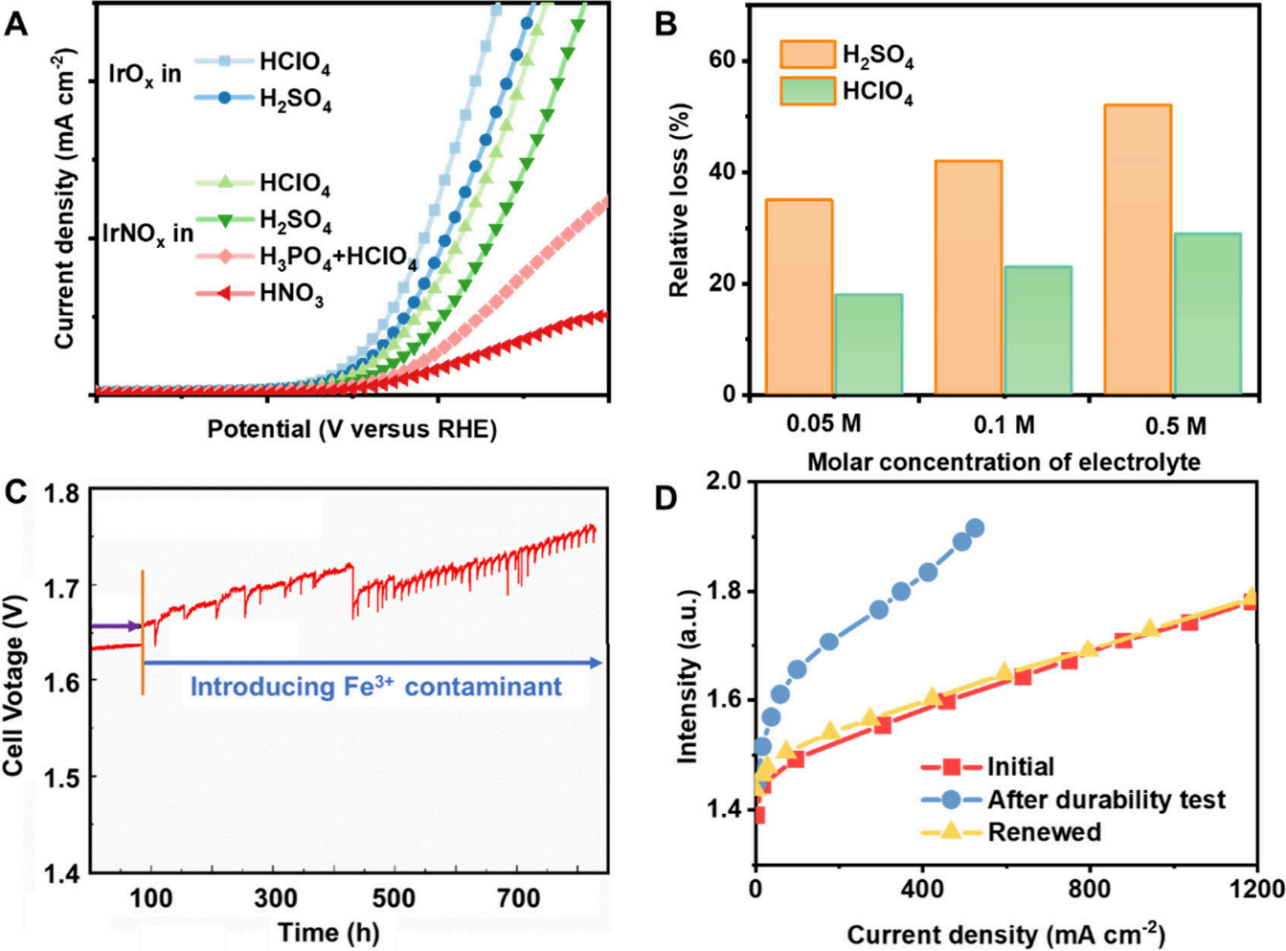
Effect of electrolyte, pH, and impurities on OER activity and stability. (A) OER polarization curves of Ir-based catalyst in H_2_SO_4_ and HClO_4_, H_3_PO_4_, and HNO_3_. Data were adapted with permission from refs (37) and (38), Copyright 2015 Royal Society of Chemistry and Copyright 2014 American Chemical Society. Note: The data presented performance trends only. (B) Relative loss of mass activity @1.55 V before and after durability test of Ir-based catalyst in different electrolytes (Durability test: CV cycling between 1.0 to 1.6 V). Adapted with permission from ref (35), Copyright 2019 Wiley. (C) *V*–*t* curve after Fe^3+^ contaminant is introduced (in MEA). Adapted with permission from ref (47), Copyright 2019 Elsevier. (D) Catalyst performance before and after durability test, and the renewed polarization curve. Adapted with permission from ref (50), Copyright 2014 Elsevier.

Schuhmann et al. demonstrated that the H_2_SO_4_ decreased the initial OER activity of Ir oxide as compared to the HClO_4_ electrolyte (Figure 1A),^37^ consistent with other reports.^35^ Furthermore, Koper et al. broadened the list of possible acidic electrolytes and discovered that Ir-coordination catalysts (IrCNO_*x*_) exhibited superior OER activity in HClO_4_ than either H_2_SO_4_ or H_3_PO_4_ or HNO_3_ (Figure 1A). This observation was attributed to the strong adsorption/poisoning effects of anions on Ir-based active sites.^38^ The above results show that the choice of electrolyte is not freewheeling, even if the catalyst is not metallic. The choice of electrolyte species should be considered in the material screening process, which is also expected to be true during the stability/durability tests.

Escudero-Escubrano et al. clearly demonstrated that the OER stability is dependent on the electrolyte, even if the H^+^ concentration is the same (e.g., 0.05 M H_2_SO_4_ and 0.1 M HClO_4_, Figure 1B).^35^ This suggests the significant effects of anions in the electrolyte on the catalyst degradation. On the other hand, toward the same electrolyte as shown in Figure 1B, we can see the concentration also affects the degradation, likely related to the different pH values.

### 2.2 Contaminants/Impurities

In addition to the type of chemical used to prepare the electrolyte, the purchased chemicals are not absolutely pure and usually used without further purification. Therefore, the effects of existing contaminants and impurities should be confirmed, assuming the used glass instruments have been thoroughly cleaned (referring to the recommendations in refs (39) and (40)).

Prior research has demonstrated that the presence of diverse impurity cations in the electrolyte significantly influences the OER catalytic performance of various transition oxide materials, including IrO_*x*_.^35,41−43^ Among them, the Fe ions are the most focused ones because most commercial chemicals contain trace amounts of iron impurities. Extensive research since the 1980s has revealed the great influence of Fe impurities (or Fe doping, even at ppb level) on enhancing the OER activity of Ni and Co hydroxides or hydroxyl oxides.^44−46^ Meanwhile in acidic media, Durand et al. observed the corrosion of steel tubes in the electrolytic cell system in 1996,^45^ which may serve as one of the factors contributing to the rise in ohmic resistance.

The introduction of Fe^3+^ ions at the 1 ppm level led to a rapid deterioration in PEM electrolyzer performance (Figure 1C), as reported by Kær et al.^47^ This deterioration was attributed to the occupation of ion exchange sites within PEM as well as the active sites by Fe ions. Consequently, there was a significant increase in charge transfer and mass transfer resistance over time.^47^ On the other hand, the Fe^3+^ ions often trigger the Fenton effect, leading to the formation of free radicals and/or reactive oxygen species (ROS), which further attack the PEM and catalysts.^47,48^ The study by Fernsch et al. further suggested that at lower current densities (<0.5 A cm^–2^), the PEM electrolyzer degraded faster, possibly due to the production of abundant H_2_O_2_ at decreasing current densities.^48^ The intrinsic damage is not recoverable, but it is possible to explore self-healing materials, i.e., PEM, to address this issue as we did recently.^49^ If the performance degradation is attributed to active site occupation in the mechanism, it is usually recoverable. For example, the performance of MEA is almost completely recovered after washing the post-mortem MEA in 0.5 M H_2_SO_4_, as shown in Figure 1D,^50^ and other reports also found a similar phenomenon.^51−54^ In addition to Fe ions, the other contaminants, e.g., Ti, Cu, Ca, etc., which may came from the feedwater, other sources such as the water tank/the piping, or be released by degraded porous transport layer (PTL), probably also lead to MEA performance degradation.^50,55^

Based on the above discussion, incorporating a circulating pump and ion purification column may be necessary to obtain accurate degradation information on the OER catalysts. At the same time, using less stainless steel pipes (plastic pipes may be better) may be efficient to avoid Fe ion contamination caused by iron corrosion. On the other hand, the utilization of Ti-PTL with Pt or Ir coating can largely avoid Ti contamination and eliminate ohmic resistance raise as the traditional PTL.^55−57^

### 2.3 Electrode Substrate Material

**Figure 2:**
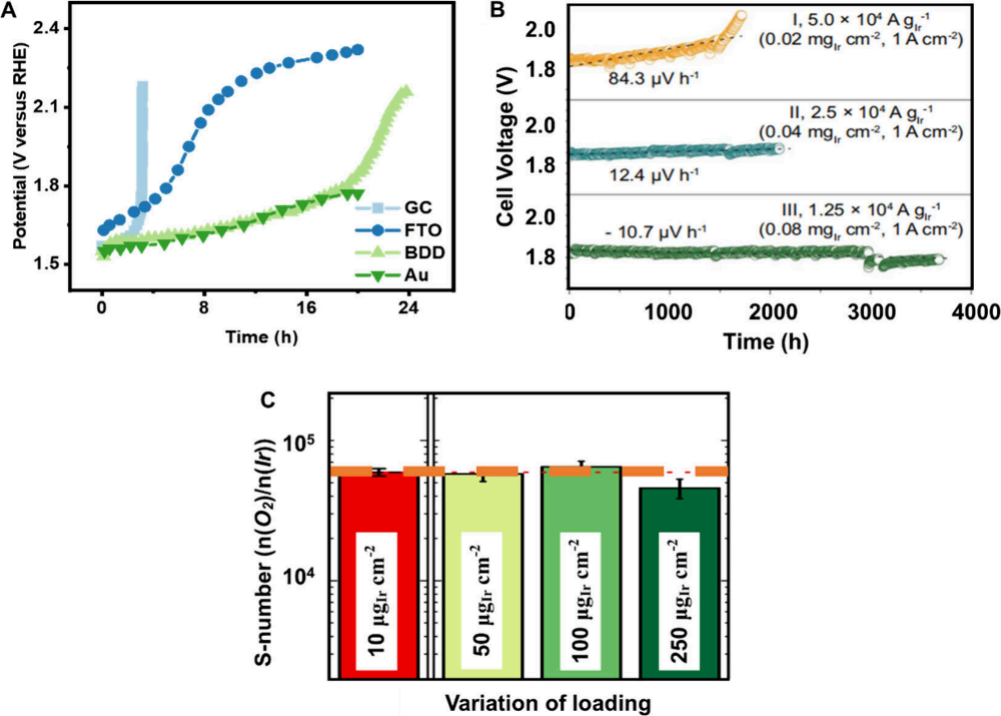
Influence of electrode substrate materials and catalyst loading on OER stability. (A) Potential evolution over time at a constant current density of 0.1 A mg^–1^_Ir_ using different electrode substrate materials. Adapted with permission from ref (59), Copyright 2017 Wiley. (B) The *V*–*t* curves using different PGM catalyst loadings. Adapted with permission from ref (67), Copyright 2024 The American Association for the Advancement of Science. (C) S-numbers of IrO_*x*_ catalyst with a variation of loadings. Adapted with permission from ref (68), Copyright 2021 The authors. Published by Springer Nature.

Different substrates have been used to prepare the working electrode during OER experiments, including glassy carbon (GC),^22,58−60^ gold,^19,59,61,62^ and boron-doped diamond,^59^ as well as antimony-, fluorine-, and indium-doped tin oxide (ATO, FTO, ITO)^24,26,63,64^ and carbon paper.^20,65,66^ In lab experiments, GC substrates are the most commonly used ones. However, the obtained results may be indefensible. The carbon as a substrate suffers from oxidation at potentials over 1.0 V, so it is actually not suitable for the OER tests at a much higher potential range. For example, the increase of overpotential @ 10 mA cm^–2^ during OER stability tests is attributed to not only the catalyst degradation but also the GC surface passivation (i.e., elevated contact resistance).^33^ Besides, the doped tin oxides (ATO, FTO, ITO) exhibit instability at high OER potentials in some publications and are not recommended as well (Figure 2A).^33,63^

In addition to the elevated contact resistance originating from the substrate itself passivation, the porous feature of thin catalyst film on the substrate could lead to the isolation of active sites by accommodating bubbles, as reported by Gasteiger et al., which was independent of any degradation or decay of the catalyst itself.^33^ Even performing the stability tests under the rotating speed of 2500 rpm, the impact of bubbles cannot be mitigated.^33^ Therefore, more resistant substrates against corrosion such as gold and boron-doped diamond, as well as dense catalyst film, are strongly recommended, particularly for stability study in two/three-electrode systems.

### 2.4 Catalyst Loading

Various catalyst loadings are applied in the stability/durability study. Actually, the catalyst loading influences the results, especially in the MEA tests. For example, a higher catalyst loading (i.e., mg_Ir_ cm^–2^) demonstrates an enhanced lifetime of PEM electrolyzers (recording voltage change at the constant current curve, *i*–*t*), as illustrated in Figure 2B.^67^ Essentially, the catalyst loading determines the metal dissolution rate per unit time (i.e., μg_Ir_ h^–1^). With regard to the degradation of the catalyst, Cherevko et al. demonstrated that there is a minimal discrepancy in the Ir dissolve rate across varying loadings (Figure 2C).^68^ Therefore, when evaluating the OER stability, it is necessary to distinguish between the metal dissolve amount and the current/voltage change in the polarization curve.

## 3 Broadened Parameters Related to Degradation

The OER catalysts need to be used in catalyst layers, for example, in the anode of MEAs. Actually, the performance and lifetime of MEAs are more helpful because the results are closer to those of real-world electrolyzer stacks/systems than the RDE setup. In this regard, other parameters related to the MEA degradation are pivotal as well, even though these parameters are not solely determined by the catalyst itself. In MEAs, the anode catalyst layer (ACL) is tightly fabricated between PEM and porous transport layer (PTL), so that the uneven stress distribution may damage ACL and lead to performance degradation. That is, the degradation of the ACL under the MEA operating conditions cannot be ignored, which is likely determined by the structure of ACL, as well as the interface engineering between PTL/ACL and PEM/ACL.

**Figure 3:**
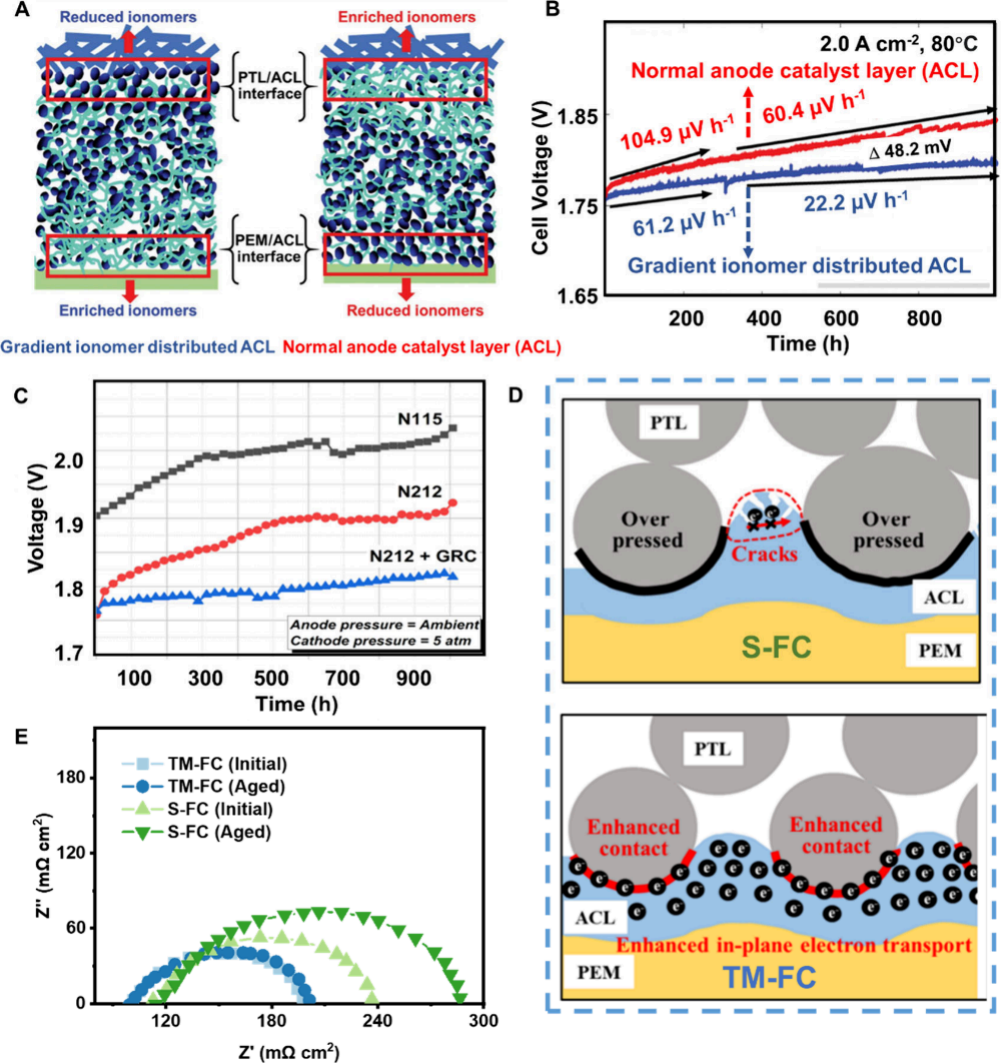
Various broadened experimental parameters related to MEA degradation. (A) Schematic diagram of ionomer distribution in gradient ionomer distributed (GID)-ACL and Norm-ACL. (B) Durability test of normal ACL and gradient ionomer distributed ACL.^69^ (A, B) Adapted with permission from ref (69), Copyright 2024 Wiley. (C) Influence of membranes on OER stability.^70^ Copyright 2024, U.S. DOE. (D) Structural schematic of ACL corresponding to the S-FC/TM-FC after durability testing. (E) Nyquist plots obtained at 1.5 V of initial and aged TM-FC, as compared to S-FC. (D, E) Adapted with permission from ref (71), Copyright 2024 The Authors. Published by American Chemical Society.

The ionomer for proton conduction is necessary in the ACL, the distribution of which affects the ACL structure. By optimizing the ionomer distribution in the ACL, i.e., the gradient ionomer distribution from low to high within ACL from the PTL/ACL to PEM/ACL interface (Figure 3A) can remarkably improve the MEA lifetime (Figure 3B, blue line).^69^ Besides, Mukundan et al. found that the MEAs using different PEMs presented different degradation behaviors. To be specific, the MEA using N212 membrane containing gas recombination catalyst (GRC) exhibited a slower voltage raise than those using N212 and N115, as illustrated in Figure 3C.^70^ The improvement in lifetime is not only due to the enhanced stability of the membrane itself but also likely attributed to the well-engineered PEM/ACL interface, which needs future studies. In addition, the well-designed flow channels in PTL could benefit lifespan by reducing the stress distribution inside the cell, e.g., applying a commercial gradient Ti-mesh instead of the traditional serpentine channels (Figure 3D), decreasing the high frequency resistance and charge transfer resistance (Figure 3E).^71,72^

## 4 Gaps between Aqueous RDE System and MEA Electrolyzer

**Figure 4:**
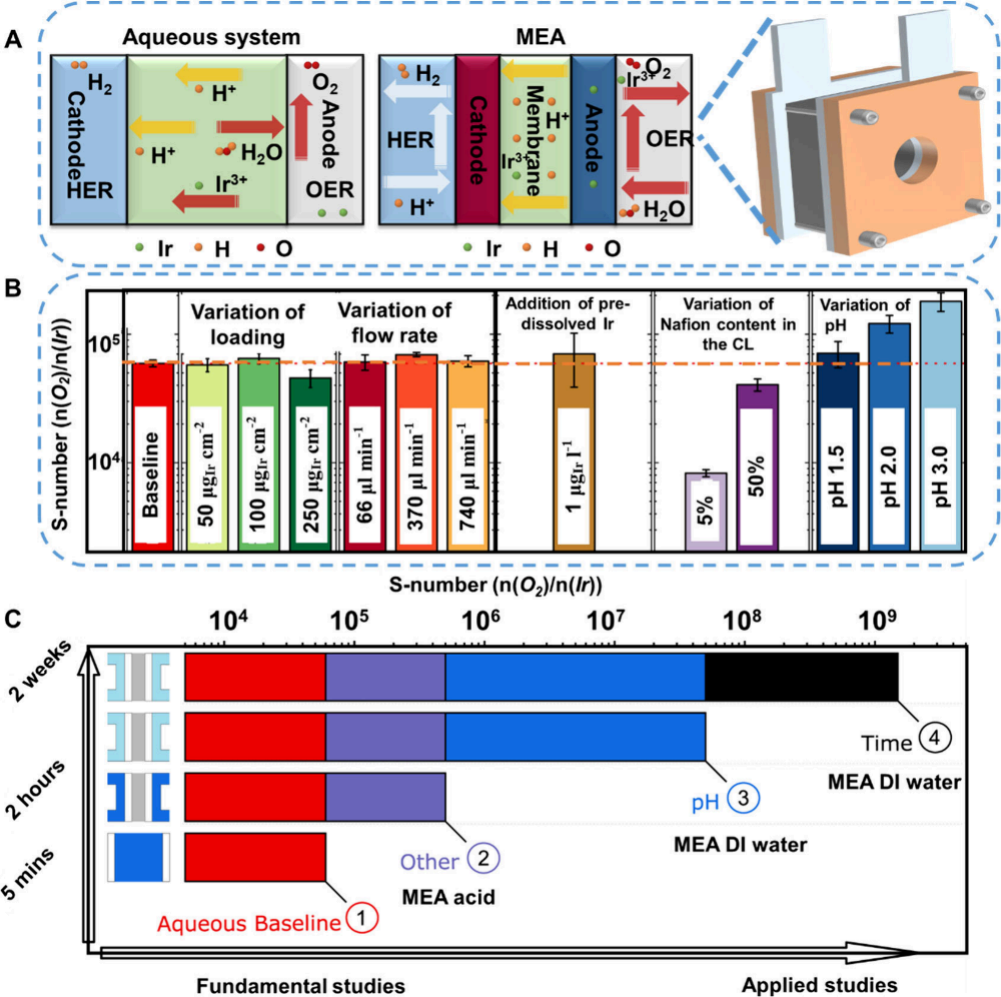
Gaps between aqueous RDE system and MEA/electrolyzer. (A) The schematic degradation mechanisms of traditional aqueous system (mainly referring to three/two electrode system) and MEA. (B) S-numbers of IrO_*x*_ catalyst under various working conditions, as measured by SFC-ICP-MS. (C) Scheme on the proposed main contributors to the dissolution discrepancy. (B, C) Adapted with permission from ref (68), Copyright 2021 The authors. Published by Springer Nature.

As mentioned above, the MEA is an integrated system involving various components such as a catalyst, ionomer, membrane, PTL, etc. Although MEA is more representative of the real operating environment, its degradation is much more complicated than the aqueous two/three-electrode system (denoted as aqueous model system) (Figure 4A). This leads to gaps in translating the aqueous model system results into MEA.^73^ For example, Ir dissolution is one of the primary degradation mechanisms of Ir-based catalysts, but the Ir dissolution rates are quite different by the aqueous model system and MEA tests. Cherevko et al. defined a S-number = *n*(O_2_)/*n*(Ir),^68,74^ reflecting the Ir dissolution rate, and studied various parameters’ effects on the S-number (Figure 4B). Among them, Figure 4B-① represents the S-number using a benchmark aqueous model system. In particular, by comparing two MEA systems using two different feed flowing over a short time (2h), i.e., acid or pure water, there is a difference of 2 orders of magnitude in the S-number between them (Figure 4B-② vs Figure 4B-③), which indicates that the pH is one of the most crucial factors leading to the gaps between RDE and MEA. With the extension of the test time, the S-number is further increased (Figure 4B-④ vs Figure 4B-③), implying that the Ir dissolution of MEA system is slowed down with longer operation, which further increases the gap between RDE and MEA results.^68^

Even though MEA tests are representative, it should be stated that the two-/three-electrode tests have their own essentiality and convenience. For example, it is targeted to reach over 80,000 h in lifetime for wide deployment, which, however, is impossible to be actually performed during material innovation.^4,75^ So far, there is still lack of clear relationships between the RDE and MEA results, but it is recommended to developing test standards using the gas diffusion electrode (GDE) as an alternative,^76−80^ which has the best of both RDE and MEA. Note that this is an eclecticism—the evaluation in real-world PEM electrolyzer is always recommended, if condition permits, to bridge the materials innovation and their applications.

## 5 Test Protocols to Accelerate Degradation

In 2013, Jaramillo et al. employed a protocol to evaluate the stability of OER catalysts, i.e., chronopotentiometry at an apparent current density of 10 mA cm^–2^ using RDE setup,^81^ which has been widely adopted by numerous research groups. The results can be regarded as a comprehensive stability for the OER catalysts because the underlying degradation mechanisms are coupled, including catalyst site deactivation, matrix corrosion, substrate surface passivation, etc. To understand the degradation mechanisms clearly, the desired test protocols are required.

On the other hand, evaluating the stability of OER catalysts across their entire lifespan under a real-world environment is not feasible, given that the real-world PEM electrolyzers can be 20,000 h (i.e., over two years),^81,82^ while the targeted lifetime is up to 80,000 h.^4^ The accelerated stress testing (AST) protocols for ORR electrocatalysts have been well agreed upon,^31,32,83^ but that for OER catalysts is still unsatisfactory.^27^

**Figure 5:**
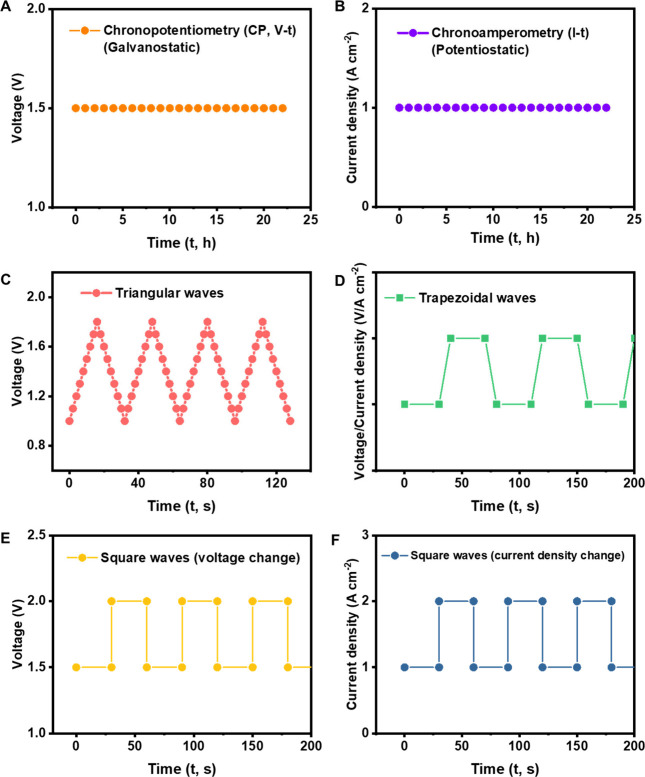
A variety of stability testing protocols. (A) Chronopotentiometry. Maintained at a constant current density, e.g., 1 A cm^–2^, to monitor real-time changes in potential/voltage. (B) Chronoamperometry. Maintained at constant potential/voltage, e.g., 1.5 V, to monitor real-time changes in current density. (C) Triangular wave. One of the most promising AST protocols in three-electrode/two-electrode systems. (D) Trapezoidal wave. A promising AST protocols in MEA/electrolyzer systems. (E) Square wave. Potential/voltage “step” changes. (F) Square wave. Current density “step” changes.

In the past, in the ORR community, “stability” and “durability” have been defined as the ability against performance degradation under potentio-/galvano-static and potentio-dynamic conditions, respectively.^31,84^ Similarly, we also recommend distinguishing the “stability” and “durability” for the study of the OER catalysts using different protocols, as shown in Figure 5. This heterogeneity in testing protocols has rendered challenging comparative analysis of stability/durability across different groups. Consequently, it is imperative to engage in discussions and explorations to develop rational and effective stability/durability testing protocols.

Compared to current density-controlling tests, the potential/voltage is more selected parameters under control. The test protocol for the OER should be learned from the ORR study. In a standard square wave to accelerate ORR catalyst degradation, the Upper Potential Limit (UPL) and Lower Potential Limit (LPL) are recommended as 0.95 and 0.6 V, respectively, by U.S. DOE.^32^ Given that the instability and degradation primarily originate from high potential, the selection of UPL is important in designing test protocols.

**Figure 6:**
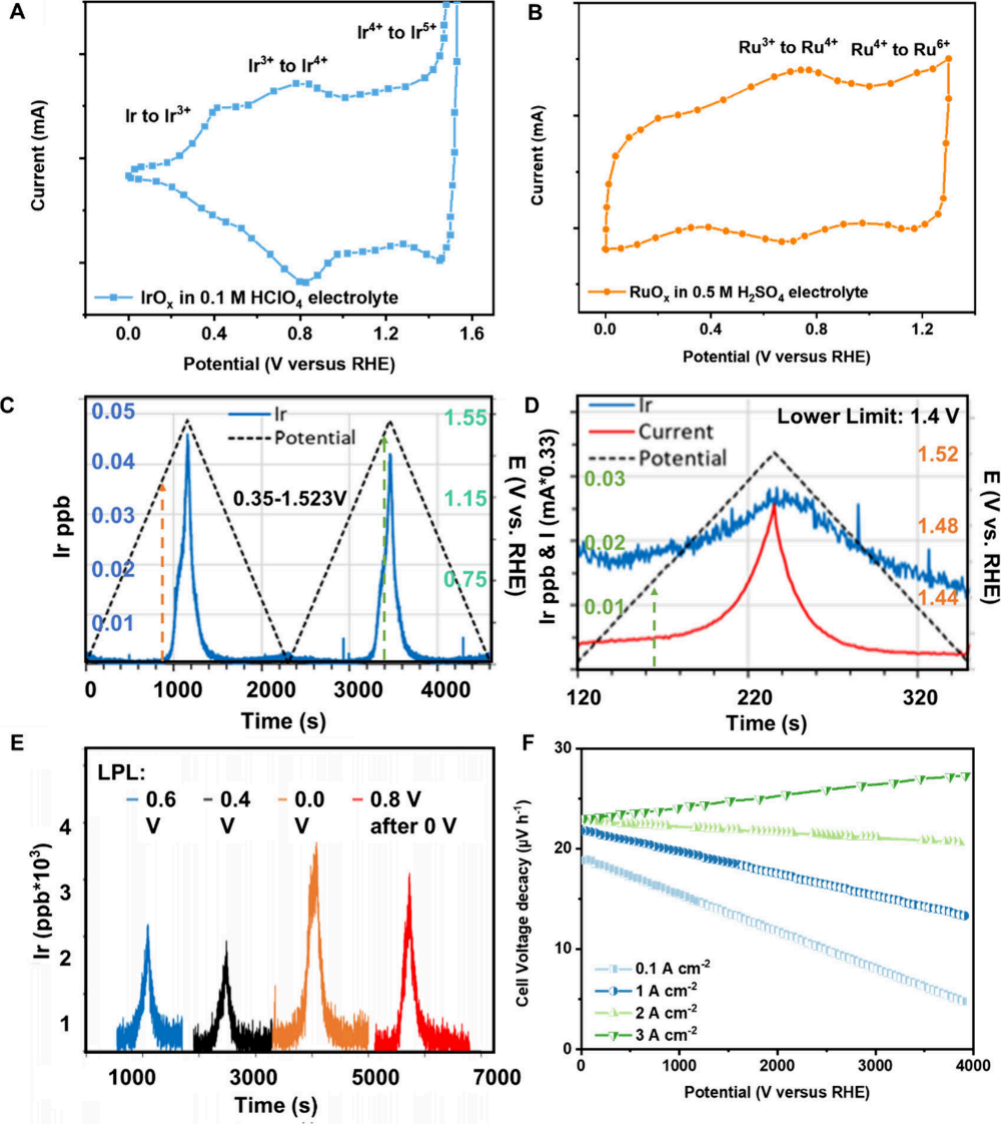
Indicators related to LPL and UPL. (A) Typical IrO_*x*_ CV curve.^85^ Copyright 2023, U.S. DOE. (B) Typical RuO_*x*_ CV curve.^86^ Adapted with permission from ref (86), Copyright 2023 Wiley. (C) Ir dissolution between 0.35 and 1.523 V.^87^ (D) Ir dissolution and OER current curve between 1.4 and 1.523 V.^87^ (C, D) Copyright 2023, U.S. DOE. (E) Ir dissolution curve of various LPL.^70^ (F) Cell voltage decay rate at various current densities.^70^ (E, F) Copyright 2024, U.S. DOE.

We note that with the increase of the various highly stable OER electrocatalysts reported in the literature, clear classification criteria must be established for these materials. We propose to classify the OER electrocatalysts into PGM and PGM-free sections, as in the already widely accepted ORR community. The PGM moieties mainly include Ir/RuO_*x*_, while the PGM-free mainly include Mn/CoO_*x*_-based electrocatalysts. It should be noted that the establishment of AST protocols for OER electrocatalysts may not be as straightforward as those for ORR catalysts due to the complicated redox potentials in the OER catalysts. For example, typical cyclic voltammetry (CV) curves of Ir/RuO_*x*_-based catalysts are shown in Figure 6A,B.^85,86^ The potential selection should be based on these curves of the targeted catalysts. Taking an Ir-based catalyst as an example, the onset potential of Ir oxidation from Ir^4+^ to Ir^5+^ is about 1.25 V (Figure 6A), so that the UPL less than 1.25 V, e.g., 0.35–1.25 V, does not lead to severe IrO_*x*_ degradation. Once the applied UPL is further increased, leaching starts to take place. This was confirmed by the Ir dissolution rate between 0.35 and 1.523 V as shown in Figure 6C. Not surprisingly, if raising the LPL positively over 1.25 V, e.g., 1.4 V, the Ir leaching reasonably takes place in the whole range (Figure 6D); i.e., faster degradation can be obtained. Under a constant UPL, the different LPL leads to different Ir dissolution and activity decay (Figure 6E), further highlighting the importance of potential selection for the study of the OER stability study. The applied potential is coupled with current density, so that if the current density is controlled higher, the cell voltage degrades faster (Figure 6F).^70,87^

Based on the above discussion, for IrO_*x*_-based electrocatalysts, the CV cycle between 1.2 and 1.6 V probably is one of the most reasonable options to achieve AST. Specific AST protocols for RuO_*x*_-based electrocatalysts have not been well agreed—the progress in IrO_*x*_-based catalysts could be helpful in promoting the agreement in AST protocols for RuO_*x*_-based and even PGM-free catalysts.^25,68,74,88−90^

## 6 Conclusion and Outlook

In this outlook, we briefly discuss the effects of experimental parameters and protocols on stability/durability tests for acidic OER catalysts, which have not been well agreed upon by the community, in contrast to the mature ORR fields. We are not aiming at fully understanding the degradation mechanisms of OER catalysts in such a short work but to portray the importance of future studies on reasonable stability/durability test standards and procedures.

Stability/durability studies are usually regarded as tools for characterization in most literature, which, however, overlooks its importance. Although some experimental parameters have been understood, the gaps between the two/three-electrode setup using RDE and membrane electrode assembly (MEA) still exist. For example, the HClO_4_ seems a good choice to study the degradation of catalysts on RDE, but it is far from the real local environment of MEA, assuming the perfluorosulfonated ionomers and membranes are employed. Therefore, it is reasonable to obtain intrinsic properties of OER catalysts using HClO_4_ electrolyte under optimized pH value, chemical purity, electrode substrate, and catalyst loading, but it may be difficult to be translated into real-world MEA tests, as various factors in addition to OER catalysts such as membrane, PTL,^56,57,70,91−94^ and even working temperature^70^ bear on the MEA lifetime. In this regard, strict verification of developed “stable catalysts” in MEA is necessary,^73^ unless it is unquestionable that RDE stability is consistent with MEA.

In addition, the degradation mechanisms of catalysts are complicated, including substrate passivation, metal dissolution, detachment, gas bubble accumulation, and membrane/ionomer degradation in MEAs, which are related to the applied working conditions. In this regard, deconvoluting the different contributions to performance degradation under different working conditions is pivotal. Designing pertinent, accurate, and efficient protocols to accelerate catalyst degradation could be an important direction in future research. Importantly, it is not reliable to make a decision based on limited results. That is, the stability/durability study should not be regarded as a tool for characterization but should undergo comprehensive and repetitive evaluation using approaches from different angles including but not limited to CV (cyclic voltammetry), chronopotentiometry (*V*–*t*), or chronoamperometry (*I*–*t*).

For PGM catalysts being used in commercialized devices, it is expected to reach a long lifetime (>80,000 h) under high current density (>1 A cm^–2^) to meet the practical requirements. In contrast for PGM-free catalysts, although they have reached much better stability for acidic water splitting,^12,24^ the test protocols are usually under low current density—at high power density, even the most advanced PGM-free catalyst is not ready to be applied. Thus, we recommend researchers in this field to focus on the development of PGM-based OER catalysts in terms of their longer lifetime under harsh conditions as well as the PGM-free catalysts in terms of their degradation mechanisms and material innovation directions.
